# Prevalence and Associated Factors of Depression among PLHIV in Ethiopia: Systematic Review and Meta-Analysis, 2017

**DOI:** 10.1155/2018/5462959

**Published:** 2018-02-28

**Authors:** Tadele Amare, Wondale Getinet, Shegaye Shumet, Biksegn Asrat

**Affiliations:** Department of Psychiatry, College of Medicine and Health Science, University of Gondar, Gondar, Ethiopia

## Abstract

**Background:**

Depression is a substantial contributor to the global burden of disease and affects people in all communities across the globe. Depression is the most common psychiatric problem associated with HIV/AIDS and half of all PLWHIV with depression go underdiagnosed and untreated. Psychiatric complications of HIVAIDS delay mental health services in less affluent countries. However, there is lack of study with regard to the pooled estimation prevalence of depression in PLWHIV in Ethiopia.

**Objectives:**

The aim of this systematic review and meta-analysis is to summarize the most current available evidence from 2010 to March 2017 among adult PLWHIV in Ethiopia.

**Methods:**

The team explored multiple databases searching methods including MEDLINE/PubMed, PsycINFO, Google Advance Scholar, and Google Scholar to find studies published with the data on the prevalence of depression among PLWHIV. We searched 150 research articles; of these 143 articles were excluded. Subsequently, thirteen articles were used for synthesis prevalence and four studies were included in the synthesis effect of sex on depression among PLWHIV.

**Results:**

The total of pooled estimated prevalence of depression in PLWHIV was 36.65. Estimated prevalence of depression in three studies by using CES-D was 31.19% and in six studies by using PHQ-9 was 37.91%. The remaining four studies used a single tool: Kessler-6 Scale (15.5%), HADS (41.2%), HDSQ (43.9%), and BDI (55.8%). Factors such as age, marital status, living alone, poor medication adherence, poor social support, clinical stages II and III of HIV, stigma, income, and occupation were significantly associated with depression.

**Conclusions and Recommendation:**

The pooled estimate prevalence of depression among PLWHIV was higher than that in the general population. It is better to offer special attention to these populations.

## 1. Introduction

Depression is the most common psychiatric problem associated with HIV disease. The large meta-analysis of 10 studies found that people living with HIV had twice the risk for depression than those who were at risk for HIV but were not actually infected [1]. According to the Joint United Nations Programme on HIV/AIDS (UNAIDS), as of December 1999, globally, more than 33 million people were estimated to be living with HIV/AIDS; among these, 95% were living in the developing world [2]. Depression rates for HIV-positive people are about 60%; but half of all PLWHIV with depression go underdiagnosed and untreated [3]. Depression is a substantial contributor to the global burden of disease and affects people in all communities across the globe. Nowadays, depression is estimated to affect 350 million people. The World Mental Health Survey conducted in seventeen countries found that about 1 in 20 people reported having an episode of depression in the preceding year [4]. Many people living with HIV have depression [5, 6]. About 90% of people who die by suicide have at least one psychiatric diagnosis; of these, depressive disorders are the most commonly associated with suicidal behavior [7]. Undetected mental health problems such as depression, personality disorders, cognitive disorders, and cooccurring conditions such as substance-related disorders can affect drug adherence, clinic attendance, and quality of life and can influence the outcome of disease and high-risk behaviors that increase risk of HIV transmission [8, 9]. Globally, by 2030, depression will be the leading cause of disease burden. In low- and middle-income countries, about 76% and 85% of people with severe mental disorders do not get treatment for their mental health problem [10]. The prevalence of mental problems in HIV-infected individuals is significantly higher than that in the general population [8]. Psychiatric complications of HIV/AIDS signify a significant additional burden for mental health services and professionals in less affluent countries with high HIV prevalence rates [6]. In India, the prevalence of depression among the HIV/AIDS patients was 40% [11]. The pooled prevalence of depression in PLWHIV in sub-Saharan Africa was 9 to 32% [12]. The prevalence of depression among the HIV/AIDS patients in Nigeria was 56.7% [13], in Uganda it was 47% [14], and in South Africa it was 37.6% [15]; and in Uganda, depressive symptoms in cognitive function impaired advanced HIV (53.9%) [16]. In systematic review and meta-analysis in low-, middle-, and high-income countries, the prevalence was 12.8 to 78% [17]. In France, it was 28.1% [18]; in three African countries (Kenya, Namibia, and Tanzania), it was 28% [19]; and in Black Americans it was 58% [20]. In Ethiopia, the prevalence of HIV is unevenly distributed in the geographical locations due to varying social, structural, and economic dynamics. The prevalence of HIV was 1.6% in Amhara, 1% in Oromia, 1.1% in Somalia, 1.8% in Tigray, 0.9% in SNNP, 5.2% in Addis Ababa, and 2.8% in Harari [21]. In another study, the prevalence of HIV was 1% in Harari, 5.8% in Gambela, 0.3% in SNNP, 0.6% in Oromia, 1.1% in Amhara, 0.9% in Afar, and 1.3% in Tigray (1.3%) [22]. When there is a variation in HIV prevalence, there is also a variation of depression prevalence. In Africa, the systematic review showed that factors that associated with depression among PLWHIV were receiving poor-quality health services, being female, and lack of emotional support from friends and family [23]. In Uganda, age above 50 years and being female were associated factors for depression [14].

The aim of this systematic review and meta-analysis is to condense the most current available evidence to March 2017 among adult PLWHIV in Ethiopia: (1) prevalence of depression (defined based on screening tools) and (2) factors that affect depression in PLWHIV.

## 2. Methods

### 2.1. Eligibility Criteria

**Figure 1:**
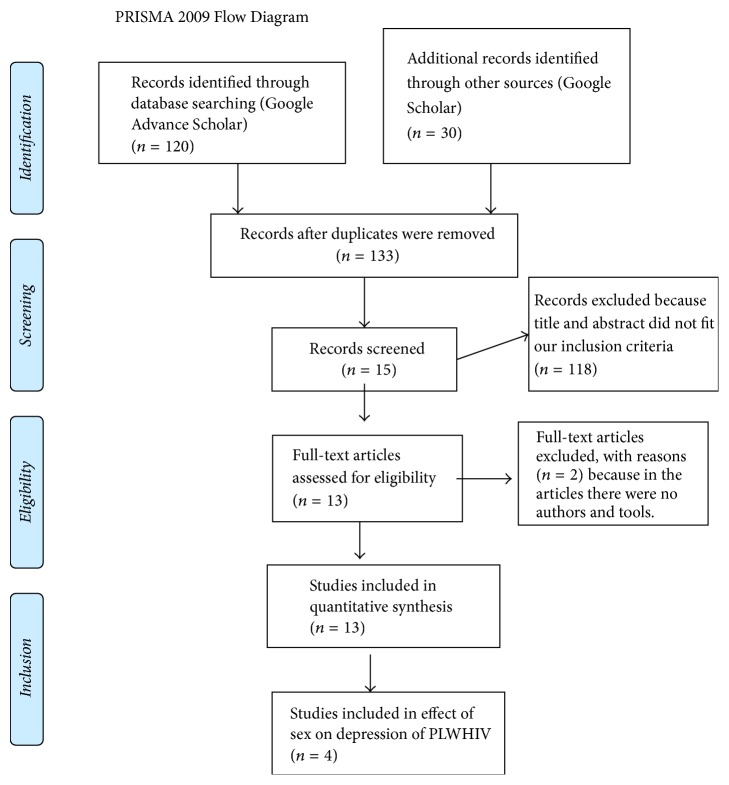
Flow chart showing how the research articles were searched (2017) [24].

The PRISMA guidelines protocol was used to write the systematic review [24]. The studies were searched in Google advance search: “depression ([MeSH Terms]) OR mental illnesses ([MeSH Terms]) AND associated factors ([MeSH Terms]) OR factors associated ([MeSH Terms]) AND HIV ([MeSH Terms]) OR PLWHIV AND Ethiopia ([MeSH Terms])” (Figure 1). The date of publication from 2010 to March 2017 and age greater than or equal to 18 years were included. Cross-sectional and longitudinal study designs were included. A comprehensive literature search was done by the terms “prevalence and associated factors of depression among PLWHIV in Ethiopia.” Mental illness and associated factors among PLWHIV in Ethiopia by Cochrane review database library and PsycINFO search strategies were also used. Figures were extracted from published reports and any lost information was gotten from investigators through email and phone.

#### 2.1.1. Inclusion and Exclusion Criteria

Literatures not written in the English language and literatures without tool to screen depression and author were excluded.

#### 2.1.2. Data Extraction and Synthesis

The assessment of each of the studies in accordance with the checklist revealed that almost all of the reports were of acceptable quality; there are eight points used to screen articles; out of these, this review document was scored 7.77 out of eight points [25]. Data were primarily appraised for quality and then extraction was made by using data extraction method. Data were analyzed using STATA V.14 statistical software. Due to the possibility of heterogeneity among the studies, random-effects meta-analysis models were preferentially reported. We developed the data extraction form that outfits the specific objective of the systematic review. It included a date of publication, the name of an author, setting, study methods, and results. Meta-analysis package was accustomed reason the pooled prevalence of depression among PLWHIV supported the tool distinction. The 1st six studies were assessed with PHQ-9 [26–31] and 3 different studies were used (CES-D) [32–34]. The remainder of studies used HADS [35], HDSQ [36], and BDI [37] and Kessler 6 scale was used to identify depression [38].

#### 2.1.3. Search Outcomes

The electronic searching of literature produced 150 articles. Among 150 research articles, 17 were excluded due to duplication and our inclusion criteria and 118 articles were excluded because title and abstract did not fit our inclusion criteria. Finally, two articles were excluded after reading full texts due to the absence of tool and author. Thirteen research articles were included to quantify the pooled estimation of depression among PLWHIV and four studies were included in effect of sex on depression (meta-analysis) (Figure 1).

#### 2.1.4. Features of Studies

All studies were conducted in Ethiopia. Eleven of them were cross-sectional surveys and the remaining two were cohort and prospective studies (Table 1).

## 3. Result

**Figure 2:**
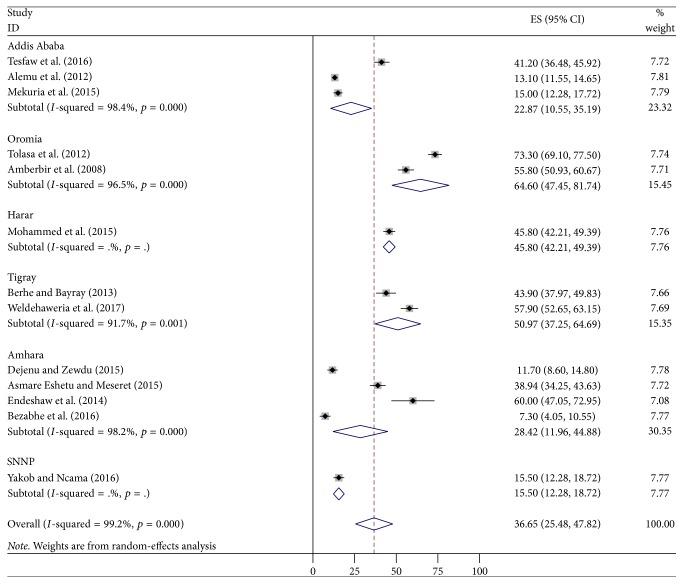
The pooled estimated prevalence of depression by region among PLWHIV in Ethiopia, 2017.

Thirteen studies were included in this study. According to different literatures in Ethiopia, the prevalence of depression ranged from 7.3% to 73.3%. The pooled estimated prevalence of depression among PLWHIV was 36.65% (95% CI: 25.48–47.82). Six regions were incorporated in this study. In Hara and Southern Nation and Nationalities of People (SNNP), a single study of each was conducted. In subgroup analysis related to region, the prevalence ranged from 15.5% (SNNP) to 64.6% (Oromia region) (Figure 2).

**Figure 3:**
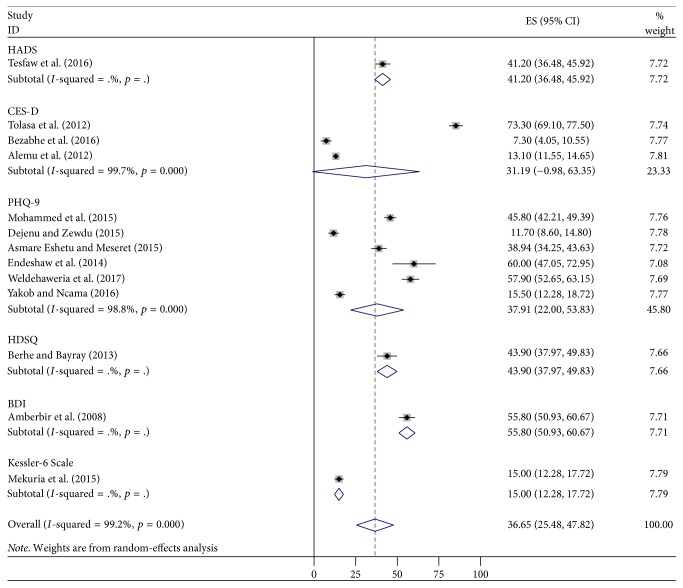
Forest plot presenting subgroup analysis of pooled estimated prevalence of depression according to the tool used among PLWHIV in Ethiopia, 2017.

The subgroup analysis of pooled estimated prevalence of depression in PLWHIV in Ethiopia according to the tool used ranged from 15.5% (Kessler) to 55.8% (BDI) (Figure 3).

## 4. Associated Factors for Depression

The factors that affect depression were different according to different studies (Table 2).

**Figure 4:**
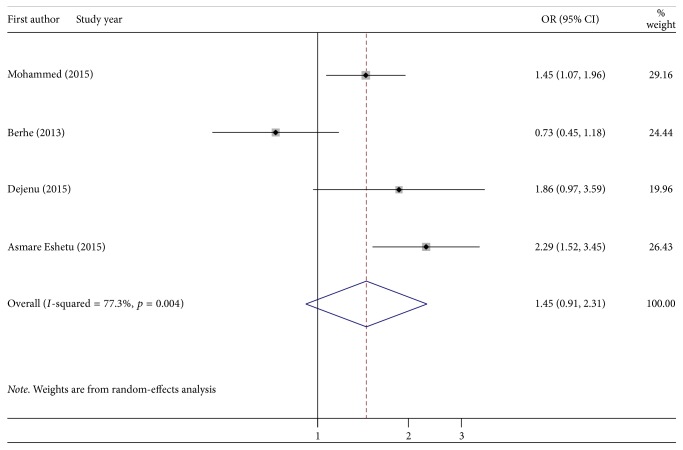
Forest plot presenting pooled random-effect size (OR) of females related to males in depressive PLWHIV patients in Ethiopia, 2017.

Is being female a risk factor for depression among PLWHIV? In four studies, being female had no significant association with depression when compared to being male (Figure 4).

## 5. Discussion

The pooled prevalence of depression symptoms in Ethiopia from 2010 to March 2017 was 36.65% (95% CI: 25.48, 47.82). The current systematic review result was almost in line with the pooled prevalence of depression in sub-Saharan African countries, 9 to 32% [12]. Our finding was similar to the finding of countries in Sub-Saharan Africa; this is due to Ethiopia being one of the countries in the Sub-Saharan Africa. The Ethiopian pooled prevalence of depressive symptoms was lower than that in the study done in Nigeria (56.7%) [13]. The difference might be due to the fact that the study done in Nigeria had a single finding, but in Ethiopia the finding was pooled. This finding was in line with the researches done in Uganda (47%) [14], South Africa (37.6%) [15], three African countries (Kenya, Namibia, and Tanzania) (28%) [19], and France (28.1%) [18]. The finding was in line with the research done in India (40%) [11]. However, this finding was lower than the result of Uganda (53.9%) [16] and Black Americans (58%) [20]. The difference might be that in Uganda the study was conducted among advanced HIV stage population and in Black Americans there are sociodemographic and tool differences. The prevalence of depression among PLWHIV in low-, middle-, and high-income countries ranged from 12.8% to 78% [17]. Our finding was also in line with this result because Ethiopia is one of the low-income countries. Based on the tool, six studies were screened by using PHQ-9 and the pooled prevalence was 37.91%, which was similar to studies that screened patients by using Center for Epidemiological Studies Depression Scale (CES-D) (31.19%) [32–34]. The reason might be that when the number of studies increases, the consistency also increases. One study indicated that the prevalence of depression in PLWHIV was 43.9% by using Hamilton's Depression Scale Questionnaire. By using Beck Depression Inventory (BDI) [37], HADS [35], and Kessler-6 Scale [38], the prevalence of depression was 55.8%, 41.2%, and 15%, respectively. In relation to regions, in Addis Ababa, three studies were conducted and the pooled prevalence was 22.87% (10.55, 35.19) [34, 35, 38], which was in line with the study done in Amhara region, 28.42% (11.96, 44.88) [27–29, 33]. This might be due to equivalent number of studies (Addis Abba = 3 studies; Amhara = 4 studies). The finding of Addis Ababa was lower than that of Oromia (64.6%) [32, 37] and Tigray region (50.97%) (37.25, 64.69) [30, 36]. The reason might be due to the fact that, in Oromia and Tigray region, two studies of each were conducted but in Addis Ababa three studies were incorporated, which means that when the number of studies increases, the precision of the study increases. The pooled prevalence of depressive symptoms in Amhara region was lower than that in the study done in Oromia and Tigray region. The possible reason might be that in Amhara four studies were included but not in the counterparts (Oromia and Tigray) and three of the Amhara region studies were assessed by PHQ-9 [27–29]. The prevalence of depression in Harar [26] and SNNP [31] was 45.8% and 15.5%, respectively. However, the finding of these two regions could not be comparable to other findings because a single study was conducted in each region.

Perceived HIV stigma and feeling stigmatized [27, 28, 35] were predictors for depression. The justification might be due to the fact that those who isolated themselves from others lead to worsening the depression. Poor social support and living alone [27, 35] are factors that affect the depressive patients, which was in line with the systematic review study in Africa [23]. The reason might be due to the fact that those who did not share their problems with other people had stress, which means that when the problems were shared with others, stress reduced by half. Poor medication adherence and last time missed any of medication were risk factors for depression in PLWHIV in Ethiopia [26, 35]. The possible reason might be due to worsening of the symptoms. Clinical stage, stage III of HIV [28, 35], and stage IV of HIV/AIDS [28] were risk factors for depression. The reason might be due to the fact that psychiatric complications of HIV/AIDS signify a significant additional burden for mental health services [6]. Patients who had low income [26, 28, 36] and experience of quitting work were significantly affected [27]. The reason might be that unfulfillment of human needs leads to depression. Being hospitalized in the past one month [28] was also another factor. The reason might be due to the fact that readmission indicates the complication of the problems. Being female was not statistically significant, 1.45 (0.91, 2.31), for depression, which contradicts with the finding of Uganda [14] and sub-Saharan Africa systematic review [23]. The possible justification was that in this finding we considered the pooled random-effect size of the four studies but in Uganda, which had a single study, and in Sub-Saharan Africa it was described qualitatively. Therefore, this finding has implication for clinicians as baseline data to link cases to psychiatry side, to reduce risks like suicide, to increase antiretroviral drug adherence, to reduce treatment delay, and to develop depression screening tool. For policymakers, it is used as baseline data to plan integrating HIV clinic with mental health and to prepare the guideline for HIV and mentally ill patients care.

## 6. Conclusion and Recommendation

The pooled estimate prevalence of depression among PLWHIV was higher than that in the general population. It is better to offer special attention to these populations.

## Figures and Tables

**Table 1:** The prevalence of depression and the tool used in each study among PLWHIV in Ethiopia, 2017.

First author	Study year	Region	Study design	Outcome	Sample size	Number of cases	Prevalence	Age (years)	Tool
Tesfaw [35]	2016	Addis Ababa	Cross-sectional	Depression	417	172	41.2	≥18	HADS
Tolasa [32]	2012	Oromia	Cross-sectional	Depression	390	299	73.3	≥18	CES-D
Mohhamed [26]	2013	Harar	Cross-sectional	Depression	740	339	45.8	≥18	PHQ-9
Berhe [36]	2012	Tigray	Cross-sectional	Depression	269	118	43.9	≥18	HDSQ
Dejenu [27]	2014	Amhara	Cross-sectional	Depression	412	48	11.7	≥18	PHQ-9
Asmare Eshetu [28]	2013	Amhara	Cross-sectional	Depression	416	162	38.94	≥18	PHQ-9
Amberbir [37]	2006-2007	Oromia	Prospective	Depression	400	223	55.8	≥18	BDI
Endeshaw [29]	2011	Amhara	Cross-sectional	Depression	55	33	60	≥18	PHQ-9
Bezabhe [33]	2012-2013	Amhara	Cohort	Depression	246	18	7.3	≥18	CES-D
Alemu [34]	2010	Addis Ababa	Cross-sectional	Depression	1815	238	13.1	≥18	CES-D
Woldehawaria [30]	2014	Tigray	Cross-sectional	Depression	340	197	57.9	≥18	PHQ-9
Yakob [31]	2015	SNNP^*∗*^	Cross-sectional	Depression	485	75	15.5	≥18	PHQ-9
Mekuria [38]	2013	Addis Ababa	Cross-sectional	Depression	664	99	15	≥18	Kessler-6 Scale

^*∗*^Southern Nation and Nationalities of People. HADS: Hospital Anxiety and Depression Scale; PHQ-9: Patient Health Questionnaire-9; CES-D: Center for Epidemiological Studies Depression Scale; BDI: Beck Depression Inventory; HDSQ: Hamilton's Depression Scale Questionnaire.

**Table 2:** Associated factors for depression among PLWHIV in Ethiopia, 2017.

Variables	Factors that affect depression in PLWHIV	First author
Sex	Being male (AOR = 1.633; 95% CI: 1.138, 2.342)	Mohammed [26]
	Being female (AOR = 2.071; 95% CI: 1.077, 3.985)	Asmare Eshetu [28]

Age	30–39 years (AOR = 2.761; 95% CI: 1.165, 6.540), 40–49 years (AOR = 3.847; 95% CI: 1.489, 9.942), 60–69 years (AOR = 19.645; 95% CI: 4.020, 95.991) compared to age 20–29 As the age increases, depression also increases	Asmare Eshetu [28]

Marital status	Being widowed (AOR = 3.128; 95% CI: 1.700, 5.757)	Mohammed [26]

Living arrangement	Living alone (AOR = 2.465; 95% CI: 1.196, 5.078)	Dejenu [27]
	Urban dwellers (AOR = 3.19; 95% CI: 1.5, 6.65) compared to rural dwellers	Berhe [36]

Social support	Poor social support (AOR = 2.02; 95% CI: 1.25, 3.27) compared to strong social support	Tesfaw [35]

Monthly income	Earning 500–1000 (AOR = 1.924; 95% CI: 1.159, 3.195) compared to >1500 birrs	Mohammed [26]
	Lower socioeconomic class (AOR = 4.43; 95% CI: 1.35, 14.58)	Berhe [36]
	Income < 200 birrs (AOR = 3.917; 95% CI: 1.559, 9.845), 201–400 birrs (AOR = 2.796; 95% CI: 1.139, 6.865), 401–700 birrs (AOR = 2.590; 95% CI: 1.058, 6.340) compared to >700 birrs	Asmare Eshetu [28]

Occupation	Unemployed (AOR = 2.74; 95% CI: 1.34, 5.57) and government employees (AOR = 3.56; 95% CI: 1.73, 7.30) compared to privately employed	Berhe [36]
	Quitting work (AOR = 2.73; 95% CI: 1.778, 6.329) compared to those in work	Dejenu [27]

Stigma and discrimination	Perceived HIV stigma (AOR = 3.60; 95% CI: 2.23, 5.80) compared to not having perceived HIV stigma	Tesfaw [35]
	Being teased, insulted, or sworn at (AOR = 2.286; 95% CI: 1.216, 4.297) Gossiped about (AOR = 2.990; 95% CI: 1.682, 5.313)	Mohammed [26]
	Stigma and discrimination from the community were 3 times more likely to have depression than their counterparts (AOR = 3.42; 95% CI: 1.628, 7.188)	Dejenu [27]
	Felt stigmatized were about 4 times (AOR = 3.597; 95% CI: 1.861, 6.954) more likely to feel stigma than the counterparts	Asmare Eshetu [28]

Medication adherence	Poor medication adherence around 2 times (AOR = 1.61; 95% CI: 1.02, 2.55) had depression compared to good medication adherence	Tesfaw [35]
	Last time missed any of medication (AOR = 5.274; 95% CI: 2.583, 10.768) compared to those who take medication regularly	Mohammed [26]

Clinical stage of HIV/AIDS	Stage III HIV/AIDS (AOR = 2.317; 95% CI: 1.108, 4.848) Stage IV HIV/AIDS (AOR = 8.769; 95% CI: 1.928, 39.872)	Asmare Eshetu [28]
	HIV stage III (AOR = 2.80; 95% CI: 1.50, 5.21) when compared to clinical stage I of HIV/AIDS	Tesfaw [35]

Hospitalization	In the past one month was (AOR = 15.262; 95% CI: 1.463, 159.219)	Asmare Eshetu [28]
